# Long‐Term Effects of Nusinersen Dosing Frequency on Adult Patients With Spinal Muscular Atrophy: Efficacy of a 6‐Month Dosing Interval

**DOI:** 10.1002/brb3.70528

**Published:** 2025-05-05

**Authors:** Keita Takahashi, Hitaru Kishida, Misako Kunii, Yosuke Miyaji, Yuichi Higashiyama, Hiroshi Doi, Naohisa Ueda, Hideyuki Takeuchi, Fumiaki Tanaka

**Affiliations:** ^1^ Department of Neurology and Stroke Medicine Yokohama City University Graduate School of Medicine Yokohama Japan; ^2^ Department of Neurology Yokohama City University Medical Center Yokohama Japan

**Keywords:** adult patient, long‐term efficacy, nusinersen, spinal muscular atrophy (SMA), survival motor neuron protein (SMN)

## Abstract

**Objective:**

Spinal muscular atrophy (SMA) is a genetic disease caused by the degeneration of spinal motor neurons due to a deficiency in survival motor neuron protein (SMN) protein, leading to progressive muscle atrophy and weakness. nusinersen, an antisense oligonucleotide that increases SMN protein expression, has shown effectiveness in both pediatric and adult patients with SMA. While it is administrated every 4 months during the maintenance period in most countries, the dosing interval is 6 months in Japan. The impact of this dosing difference on long‐term outcomes is not fully understood. This study evaluates the long‐term efficacy of the 6‐month dosing protocol of nusinersen in adult SMA patients.

**Methods:**

We assessed 14 adult patients treated with nusinersen every 6 months over a period of up to 39 months using the Hammersmith Function Motor Scale Expanded (HFMSE) and Revised Upper Limb Module (RULM). The results were compared with those from a recent cohort study of adult SMA patients in Europe.

**Results:**

For ambulatory patients, the mean changes in HFMSE scores at 15, 27, and 39 months were 6.7, 8.3, and 8.0 points, respectively. These results were similar to those observed in the European cohort. In contrast, for nonambulatory patients, the mean changes in HFSME scores were –0.3, –1.4, and –1.3 points, and the mean changes in RULM scores were 2.0, 0.5, and 1.0 points at the same time points. These results were generally less favorable compared to the European cohort but did not reach clinically meaningful deterioration.

**Discussion:**

The findings of this study suggest that the 6‐month nusinersen dosing protocol provides sustained long‐term benefits for ambulatory adult SMA patients. For nonambulatory patients, the 6‐month protocol appears less effective than the 4‐month protocol. We believe that future nusinersen treatment strategies for adult SMA patients should be flexible, with adjustments based on disease severity. In particular, increasing the dosing frequency and/or dosage in nonambulatory patients may lead to greater improvements.

## Graphical abstract

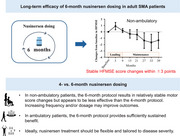

**Abbreviations**
HFMSE: Hammersmith Function Motor Scale Expanded
mRNA: messenger RNA
RULM: Revised Upper Limb Module
SMA: spinal muscular atrophy
SMN: survival motor neuron

## 1 Introduction

Spinal muscular atrophy (SMA), an autosomal recessive genetic disorder caused by the degeneration of lower motor neurons in the anterior horn of the spinal cord, is characterized clinically by progressive muscle atrophy and weakness, predominantly affecting the proximal muscles and lower limbs. The most common genetic cause of SMA is the biallelic deletion of the survival motor neuron 1 (*SMN1*) gene on chromosome 5q13.2 (5q SMA), leading to a deficiency in the full‐length SMN protein and the subsequent degeneration of motor neurons (Brzustowicz et al. 1990; Gilliam et al. 1990; Lefebvre et al. 1995; Lunn and Wang 2008). In SMA, a small amount of full‐length SMN protein is produced from *SMN2*, a paralogous gene located near *SMN1*, and the higher the copy number of *SMN2*, the milder the disease severity (Gavrilov et al. 1998; Lorson et al. 1999; Vitte et al. 2007). However, most *SMN2*‐derived messenger RNA (mRNA) transcripts undergo exon 7 skipping during splicing and are rapidly degraded (Cartegni et al. 2002; Cartegni et al. 2006). Nusinersen is an intrathecally administered antisense oligonucleotide designed to regulate the splicing of *SMN2* precursor mRNA, promoting the inclusion of exon 7 and thereby increasing the levels of full‐length SMN protein (Hofmann and Wirth 2002; Hua et al. 2011; Hua et al. 2008).

Since the development of nusinersen therapy, substantial disease‐modifying effects have been reported in pediatric SMA patients (Finkel et al. 2017; Mercuri et al. 2018). Efficacy has also been demonstrated in adult SMA patients, although most reports were based on relatively short‐term evaluations (6 to 14 months) (Coratti et al. 2022; De Wel et al. 2021; Gavriilaki et al. 2022; Hagenacker et al. 2020; Jochmann et al. 2020; Maggi et al. 2020; Vazquez‐Costa et al. 2022). A recent multinational long‐term observational study in Europe indicated that although nusinersen therapy was effective in adult patients, a continuous improvement from 14 to 38 months was not observed (Gunther et al. 2024). Meanwhile, real‐world evidence regarding the effect of the dosing frequency of nusinersen on patients with SMA remains limited. Nusinersen is administrated every 4 months during the maintenance phase in most countries, whereas in Japan, it is administered every 6 months due to national policies, resulting in a lower total dose over the same treatment duration.

In this study, we report the long‐term efficacy of nusinersen therapy administered every 6 months up to 39 months in 14 adult patients with SMA and discuss the relationship between dosing frequency and therapeutic outcomes. This report provides important evidence that may help re‐evaluate the appropriate nusinersen dosing frequency and total dose.

## 2 Patients and Methods

### 2.1 Study Design and Participants

This was a retrospective observational study conducted at two institutions (Yokohama City University Hospital and Yokohama City University Medical Center) involving adult patients diagnosed with 5q SMA. Inclusion criteria were patients with confirmed homozygous deletions of either or both of exons 7 and 8 of *SMN1*, who had received continuous nusinersen treatment for at least 9 months. All participants underwent detailed neurological assessments by neurologists and rehabilitation specialists at each hospital.

### 2.2 Procedures

**FIGURE 1:**
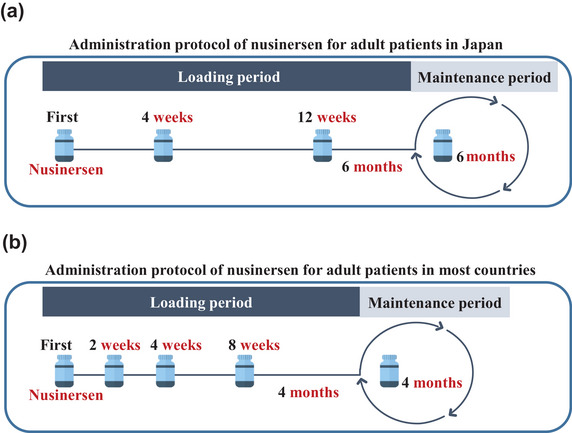
**Administration protocol for nusinersen**. (a) Japan. (b) Other countries.

After baseline evaluations, patients received an intrathecal injection of nusinersen (12 mg) on days 0, 28, and 84 as part of the loading period, followed by the maintenance period, during which the same dose was administered every 6 months (Figure 1a). For the injections, a few patients with severe scoliosis underwent lumbar puncture under the guidance of spinal computed tomography or ultrasound, whereas most patients received the standard procedure. The frequency of this treatment is lower than that of the protocol used in most countries, which involves 4 doses during the loading period and administration every 4 months during the maintenance period (Figure 1b).

We assessed 14 adult SMA patients using the Hammersmith Functional Motor Scale Expanded (HFMSE), which evaluates a wide range of motor functions across 33 items, primarily focusing on the trunk and lower limbs (O'Hagen et al. 2007). This scale is considered less sensitive to changes in nonambulatory patients (E. Mazzone et al. 2014; E. S. Mazzone et al. 2017). The assessment was conducted every 6 months up to 39 months. We also used the Revised Upper Limb Module (RULM), a 20‐item scale, to assess upper limb function. Because SMA patients typically retain more muscle strength in their upper limbs compared with their lower limbs, the RULM is considered a useful scale for assessing nonambulatory patients with SMA (Coratti et al. 2022) (E. S. Mazzone et al. 2017; Pera et al. 2019). Changes in HFMSE and RULM scores between baseline values and post‐treatment evaluations were analyzed statistically. A change of 3 or more points on the HFSME and 2 or more points on the RULM was considered clinically meaningful (Pera et al. 2017; Pera et al. 2019).

We also compared our results with those from a recently published European cohort study, which followed a 4‐month dosing schedule during the maintenance period (Gunther et al. 2024). Because of slight differences in the timing of evaluations, we compared the score changes at 15, 27, and 39 months in our study with those at 14, 26, and 38 months in the European study.

This study was approved by the Ethics Committees of Yokohama City University (approval number: B201200006), and informed consent for research participation was obtained from all patients.

### 2.3 Statistical Analysis

Statistical analyses were performed using the paired *t*‐test for related samples and the Mann–Whitney *U‐*test for group comparisons of the pre‐ and post‐treatment scores at 15, 27, and 39 months, as appropriate, using GraphPad Prism version 6.0 (GraphPad Software). A *p*‐value less than 0.05 was considered statistically significant.

## 3 Results

The characteristics of the 14 patients are summarized in Table 1. The mean age at treatment initiation was 42.8 ± 17.5 years, and 8 patients were male. Ten patients (3 with SMA type 2 and 7 with SMA type 3) were nonambulatory, and 4 patients (3 with SMA type 3 and 1 with SMA type 4) were ambulatory. The *SMN2* copy number was 2 in 1 patient, 3 in 4 patients, and 4 in 9 patients. The mean baseline HFMSE score for all patients was 21.1 points. For ambulatory and nonambulatory patients, the mean scores were 43.3 points and 12.3 points, respectively. The mean baseline RULM score for all SMA patients was 18.6 ± 11.1 points. For ambulatory patients and nonambulatory patients, the mean scores were 32 points and 14.2 points, respectively.

**TABLE 1:** Demographic and clinical characteristics of patients analyzed in this study.

	No. of patients (*n*)	Male/female	Age (years) (mean ± SD)	Baseline HFMSE (mean ± SD)	Baseline RULM^a^ (mean ± SD)	Baseline HFMSE in the European study cohort (Gunther 2024) (mean ± SD)	Baseline RULM in the European study cohort (Gunther 2024) (mean ± SD)
**All SMA patients**	14	8/6	42.8 ± 17.5	21.1 ± 19.7	18.6 ± 11.1	25.3 ± 21.7	24.0 ± 12.5
**Ambulant**	4	3/1	30.8 ± 18.1	43.3 ± 11.2	32	NA	NA
SMA type 3	3	2/1	28.3 ± 21.4	37.7 ± 1.5	27		
SMA type 4	1	1/0	38	60	37		
**Nonambulant**	10	5/5	47.6 ± 15.6	12.3 ± 14.6	14.2 ± 8.3	NA	NA
SMA type 2	3	1/2	32.7 ± 6.6	0.7 ± 1.2	4		
SMA type 3	7	4/3	54.0 ± 13.8	17.3 ± 15.0	16.2 ± 7.5		

SMA = spinal muscular atrophy; HFMSE = Hammersmith Functional Motor Scale Expanded; RULM = Revised Upper Limb Module; SD = standard deviation; NA = not available.

^a^The mean baseline of RULM scores was calculated for two ambulatory patients and six nonambulatory patients.

The mean changes in HFMSE scores from baseline for all patients were 1.5, 1.4, and 1.8 points at 15, 27, and 39 months, respectively; however, none of these changes reached statistical significance (Table 2). In contrast, the European cohort showed changes of 1.7, 1.2, and 1.5 points at 14, 26, and 38 months, which were reported as statistically significant. Additionally, the proportion of patients in our cohort showing clinically meaningful improvements was 23.1%, 25.0%, and 33.3% at 15, 27, and 39 months, respectively, which were close to those of the European cohort at the same time points.

**TABLE 2:** Changes from baseline in HFMSE scores and a comparison with those from a European study.

	Our Japanese study					A previous European study(Gunther 2024)^a^		
HFMSE score	*n*	Difference from baseline (mean ± SD)	*p*‐value^b^	Clinically meaningful improvement (%)	Clinically meaningful deterioration (%)^c^	Difference from baseline (mean± SD)	*p*‐value^b^	Clinically meaningful improvement (%)
**All SMA**								
15 months	13	1.5 ± 3.8	0.165	23.1	7.7	1.7	< 0.0001	28.7
27 months	12	1.4 ± 5.2	0.364	25.0	16.7	1.2	0.0012	28.7
39 months	9	1.8 ± 5.3	0.342	33.3	22.2	1.5	0.0002	30.0
**Ambulant**								
15 months	3	6.7 ± 3.5	0.0407	100.0	0.0	2.5 ± 4.8	< 0.0001	44.0
27 months	3	8.3 ± 1.2	0.0032	100.0	0.0	2.2 ± 5.2	0.0004	42.9
39 months	3	8.0 ± 1.0	0.0026	100.0	0.0	2.4 ± 4.6	0.0002	45.6
**Nonambulant**								
15 months	10	−0.3 ± 2.0	> 0.999	0.0	10.0	1.1 ± 3.5	0.0026	17.5
27 months	9	−1.4 ± 3.5	0.5588	0.0	25.0	0.4 ± 4.1	0.0032	17.0
39 months	6	−1.3 ± 3.1	0.2022	0.0	16.7	0.7 ± 3.9	0.0091	15.9

SMA = spinal muscular atrophy; HFMSE = Hammersmith Functional Motor Scale Expanded; SD = standard deviation.

^a^We compared the score changes at 15, 27, and 39 months in our study with those at 14, 26, and 38 months in the European study.

^b^The *p*‐value was calculated using a paired *t*‐test for the ambulant group and the Mann–Whitney *U*‐test for the nonambulant group.

^c^The ratio of clinically meaningful deterioration was not reported in the European study except for total SMA patients, where the values were 8%, 15%, and 13%, respectively at 14, 26, and 38 months, respectively [22].

In the ambulatory group, the mean changes in HFMSE scores from baseline were 6.7, 8.3, and 8.0 points at 15, 27, and 39 months, respectively, and all these changes were statistically significant. In the European cohort, the changes were 2.5, 2.2, and 2.4 points at 14, 26, and 38 months, all of which were also statistically significant. Furthermore, clinically meaningful improvements were observed in all patients in our cohort at every evaluation point, whereas approximately half of the patients in the European cohort showed such improvements during the same period.

In contrast, for the nonambulatory group, the changes in HFMSE scores from baseline at 15, 27, and 39 months were negative (−0.3, −1.4, and −1.3 points, respectively), although these changes did not reach statistical significance (Table 2). This contrasts with the European cohort, where statistically significant positive changes (1.1, 0.4, and 0.7 points at 14, 26, and 38 months, respectively) were observed. Additionally, no patients in our cohort showed clinically meaningful improvement at any assessment time points, whereas approximately 15% of the nonambulatory patients in the European cohort demonstrated clinically significant improvements during the same period. In our cohort, clinically meaningful deteriorations were observed in 10.0%, 25.0%, and 16.7% of nonambulatory patients at 15, 27, and 39 months, respectively (Table 2).

The analysis of changes in RULM scores was limited to nonambulatory patients because there were many missing values in the ambulatory group data. The mean changes in RULM scores from baseline at 15, 27, and 39 months were 2.0, 0.5, and 1.0 points, respectively, none of which reached statistical significance (Table 3). In contrast, the European cohort reported changes in RULM scores of 1.1, 1.0, and 1.2 points at 14, 26, and 38 months, respectively, all of which were statistically significant. The proportion of patients showing clinically meaningful improvements in our cohort was 33.3% at all evaluation points, which was similar to those in the European cohort.

**TABLE 3:** Changes from baseline in the RULM scores and comparison with those from a European study.

	Our Japanese study					A previous European study (Gunther 2024)^a^		
RULM score	*n*	Difference from baseline (mean ± SD)	*p*‐value^b^	Clinically meaningful improvement (%)	Clinically meaningful deterioration (%)^c^	Difference from baseline (mean± SD)	*p*‐value^b^	Clinically meaningful improvement (%)
**Nonambulant**								
15 months	6	2.0 ± 2.9	0.1275	33.3	0.0	1.1 ± 2.9	< 0.0001	37.4
27 months	6	0.5 ± 2.6	0.6560	33.3	16.7	1.0 ± 2.9	0.0007	38.4
39 months	6	1.0 ± 2.0	0.2752	33.3	16.7	1.2 ± 3.0	0.0013	36.4

RULM = Revised Upper Limb Module; SD = standard deviation.

^a^We compared the score changes at 15, 27, and 39 months in our study with those at 14, 26, and 38 months in the European study.

^b^The *p*‐value was calculated using a paired *t*‐test.

^c^The ratio of clinically meaningful deterioration was not reported in the European study.

**FIGURE 2:**
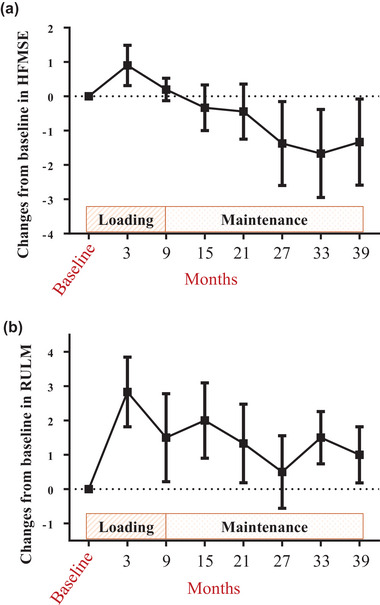
**Changes in motor function scores in nonambulatory SMA patients receiving nusinersen therapy**. (a) Changes in HFMSE scores from baseline. (b) Changes in RULM scores from baseline. The dashed horizontal lines represent the baseline values. Changes in scores at each assessment point are expressed as the mean ± standard error of the mean (SEM).

We also plotted changes in HFMSE and RULM scores from the loading period (0–9 months) to the maintenance period (10–39 months) in nonambulatory patients (Figure 2). For both scales, the maximum scores were recorded during the loading period. Subsequently, the scores decreased gradually during the maintenance period. However, during the maintenance period, the mean scores remained relatively stable, staying within the range of clinically meaningless changes compared with the baseline scores.

## 4 Discussion

The efficacy of nusinersen treatment for adult patients with SMA has primarily been documented in studies using a dosing protocol of every 4 months during the maintenance period. However, there is limited evidence regarding the long‐term efficacy of the protocol with dosing every 6 months.

The characteristics of our cohort were similar to those of the European cohort, with a relatively large proportion of nonambulatory SMA type 2 and 3 patients (Gunther et al. 2024), although the baseline HFMSE scores were slightly lower in our cohort. In all patients, the mean changes in HFMSE scores from baseline at 15, 27, and 39 months were similar to those observed in the European cohort at 14, 26, and 38 months (Table 2). Moreover, the proportion of patients showing clinically meaningful improvements in HFMSE scores was 33.3% at 39 months in our cohort and 30.0% at 38 months in the European cohort, with no notable differences between the two cohorts.

In the nonambulatory group, however, the mean changes in HFMSE scores from baseline at 15, 27, and 39 months were negative at all time points examined (Table 2, Figure 2a), in contrast to the statistically significant positive changes observed in the European cohort. Additionally, the proportion of patients showing clinically meaningful improvements in HFMSE scores was lower in our cohort (Table 2). Of note, even during the maintenance period, the average change in HFMSE scores did not reach the threshold for a clinically meaningful deterioration in function (i.e., a decrease of 3 points) (Figure 2a). Furthermore, although the mean change in RULM scores was not statistically significant, it showed a trend toward improvement (Table 3, Figure 2b), which was similar to that observed in the European cohort. Therefore, although the efficacy of the 6‐month dosing protocol may be somewhat inferior to that of the 4‐month protocol for nonambulatory patients, it can still be considered clinically effective over the long term.

In Turkey, the 4‐month protocol is followed during the maintenance period; however, the dosing protocol during the loading phase is the same as in Japan (Figure 1a). Thus, whereas the effects of 6 doses are reflected in motor function at 14 months in most countries, the effects of only 4 doses are observed at 15 months in Japan and Turkey. A study conducted in Turkey reported that nonambulatory patients showed a 2‐point improvement in the HFMSE score for SMA type 2 and a 4‐point improvement for SMA type 3 at 15 months (Arslan et al. 2023). These results were better than those observed in all nonambulatory patients in our cohort (−0.3 points) and the European cohort (1.1 points). This difference may be because of the higher baseline HFMSE scores in the Turkish cohort's nonambulatory patients (5 points for SMA type 2 and 29 points for SMA type 3) compared with those in our cohort (0.7 points for SMA type 2 and 17.3 points for SMA type 3). Thus, the effect of nusinersen may largely depend on the baseline HFMSE score rather than the number of doses administered. Indeed, patients in our cohort with higher baseline HFMSE scores—specifically, ambulatory patients—showed statistically significant improvements in HFMSE scores from baseline at all three evaluation points during the maintenance period, despite the lower dosing frequency. The proportion of patients showing clinically meaningful improvements was also high, and these results were comparable with those observed in the European cohort (Table 2). Therefore, the 6‐month dosing frequency of nusinersen appears to be adequately effective in adult ambulatory SMA patients.

This study has an inherent limitation because of its small sample size, which may not be sufficient to detect statistically significant differences. Additionally, only the HFMSE and RULM scales were used to analyze motor function, and other evaluations, such as the 6‐minute walk test, were not included because of the small number of cases in which they were performed. Nevertheless, this study demonstrated the long‐term efficacy of the 6‐month nusinersen maintenance dosing protocol. In particular, for ambulatory patients, the efficacy was not inferior to that of the 4‐month maintenance dosing protocol. Considering the invasiveness of intrathecal injections and the burden on patients, the 6‐month dosing interval seems reasonable.

Regarding the pharmacokinetic properties of nusinersen, data from previous clinical trials in pediatric SMA patients have shown that higher doses and/or more frequent dosing during the loading period resulted in higher cerebrospinal fluid (CSF) concentrations (Finkel et al. 2022). Pharmacokinetic/pharmacodynamic modeling predicted that when the steady‐state CSF concentration increases from 5 ng/mL (achieved with the 12 mg dose) to 12 ng/mL, the score on the Children's Hospital of Philadelphia Infant Test of Neuromuscular Disorders, a motor function scale for children, will increase by an additional 5.2 points (Finkel et al. 2022). Accordingly, higher doses of nusinersen are expected to demonstrate even greater efficacy compared with the approved 12 mg dose (Finkel et al. 2022). More frequent dosing also leads to higher CSF concentrations, potentially yielding effects similar to those achieved with higher doses.

Considering these findings, for nonambulatory patients, especially those with low baseline HFMSE scores, increasing the dosing frequency to every 4 months and/or using a higher dose during the maintenance period may enhance the therapeutic effect of nusinersen and result in better improvements in motor function.

Currently, the DEVOTE study (NCT04089566) is investigating a high‐dose regimen of 28 mg nusinersen every 4 months (Finkel et al. 2023), with an interim report indicating that patients receiving this regimen showed significant improvements compared with those in the 12 mg group (Biogen, September 4 2024). In the future, we expect that more effective nusinersen dosing protocols will be adopted globally, including in Japan, and that flexible treatment strategies will be implemented, such as adjusting dosing frequency and dosage based on the severity of the patient's condition.

## 5 Conclusion

In conclusion, we report that the long‐term efficacy of a 6‐month dosing protocol of nusinersen in adult SMA patients, which differs from the 4‐month protocol in most countries. Though it provides sustained long‐term benefits for ambulatory adult SMA patients, our data suggest that increasing the dosing frequency and/or dosage may lead to further improvements for nonambulatory patients. In the future, nusinersen treatment strategies for adult SMA patients should be flexible, with adjustments based on disease severity.

## Author Contributions

K.T. and F.T. designed the study and analyzed data. K.T., H.T., H.K., M.K., Y.M., Y.H., H.D., and N.U. treated patients and evaluated their motor functions. H.T. and H.K. supervised the data analysis and interpretation. K.T. and F.T. managed clinical data. K.T. wrote the paper. F.T. edited the manuscript.

## Ethics Statement

This study was approved by the ethics committee of the Yokohama City University Hospital (approval number: B201200006). Informed consent was obtained from each participant according to the research ethics of our university hospital.

## Conflicts of Interest

The authors declare that they have no competing interests.

### Peer Review

The peer review history for this article is available at https://publons.com/publon/10.1002/brb3.70528

## Data Availability

The data that support the findings of this study are available from the corresponding author upon reasonable request.
